# A Randomized Controlled Study of Caudal Dexmedetomidine for the Prevention of Postoperative Agitation in Children Undergoing Urethroplasty

**DOI:** 10.3389/fped.2021.658047

**Published:** 2021-09-29

**Authors:** Weichao Zhu, Jie Sun, Jianhua He, Wangping Zhang, Meng Shi

**Affiliations:** ^1^Department of Pediatric Surgery, The Affiliated Hospital of Medical School, Ningbo University, Ningbo, China; ^2^Department of Urology, Shanghai Children's Medical Center, Shanghai Jiao Tong University School of Medicine, Shanghai, China; ^3^Diagnosis and Treatment Center of Pelvic Floor, The Affiliated Hospital of Medical School, Ningbo University, Ningbo, China; ^4^Department of Anesthesiology, Women and Children's Hospital of Jiaxing University, Jiaxing, China; ^5^Department of Anesthesiology, Xuzhou Medical University, Xuzhou, China

**Keywords:** dexmedetomidine, caudal block, postoperative agitation, children, general anesthesia

## Abstract

**Background:** Postoperative agitation is a common complication in children undergoing general anesthesia. This study aimed to investigate the effect of caudal dexmedetomidine for the prevention of postoperative agitation in children undergoing urethroplasty.

**Materials and Methods:** Eighty children were prospectively recruited to this study and randomized to two groups (40 cases in each group), specifically, a dexmedetomidine group (group D) who received 0.2% ropivacaine + 0.5 μg/kg dexmedetomidine for caudal block, and a control group who received 0.2% ropivacaine alone. The time to wake up, the time to discharge from the postanesthesia care unit (PACU), the duration of the caudal block, and the Ramsay sedation scale (RSS) were evaluated in the patients. Adverse events such as postoperative agitation, respiratory depression, bradycardia, hypotension, excessive sedation, nausea, and vomiting were also recorded during the first postoperative 24 h.

**Results:** The incidence of postoperative agitation was lower in group D compared with patients in the control group (2.5 vs. 22.5%, *p* = 0.007). The time to wake up and the time to discharge from PACU were longer in group D than in the control group (15.2 ± 2.6 vs. 13.4 ± 1.3 min, 48.2 ± 7.7 vs. 41.5 ± 8.0 min, respectively, *p* < 0.001). However, the extubation times were similar between the two groups. The duration of the caudal block was longer in group D compared with the control group (8.8 ± 1.6 vs. 4.6 ± 0.7 h, *p* < 0.001).

**Conclusions:** Caudal dexmedetomidine prolongs the duration of caudal block and decreases the incidence of postoperative agitation in children undergoing urethroplasty.

**Clinical Trial Registration:** ChiCTR1800016828.

## Introduction

Postoperative agitation is one of the common complications in pediatric patients after general anesthesia (1, 2). It is characterized by crying, shouting, screaming, non-purposeful restlessness, and disorientation (3). The rate of postoperative agitation has been reported to range from 10 to 80% in pediatric patients (4).

Dexmedetomidine is an α_2_ adrenergic agonist that is used for sedation by intravenous infusion. Studies have shown that intravenous dexmedetomidine can reduce the incidence of postoperative agitation in pediatric patients receiving general anesthesia (5, 6). Also, venous infusion of dexmedetomidine may lead to delayed discharge from the hospital (7). Ropivacaine (0.2%) with or without adjuvants is usually used for the caudal block in children. However, studies on the use of caudal dexmedetomidine to prevent postoperative agitation are yet to be reported in the literature. The purpose of this study was to investigate the efficacy of caudal dexmedetomidine in reducing postoperative agitation in children undergoing urethroplasty.

## Materials and Methods

This study was conducted in accordance with the Declaration of Helsinki and approved by the Ethical Committee of the Jiaxing Children's Hospital (approval number: 2018-36, Chairman: Prof L. Xia). Written informed consent was obtained from the parents or guardians of the children recruited to the study (www.chictr.org.cn, registration number: ChiCTR1800016828).

From July 2018 to July 2019, a total of 80 children undergoing urethroplasty with ASA I–II who weighed between 10 and 30 kg and were aged 1 to 6 years were recruited to this study. Children with cardiopulmonary diseases, body mass index (BMI) >29 kg/m^2^, and contradictions to caudal block were excluded from the study. Children were randomized to the control group or the dexmedetomidine group (the group D) with 40 patients in each group. The anesthesiologists, nurses, investigators, and children were blinded to the allocated groups.

All children were fasted for 6–8 h before treatment and had no premedications. Upon arrival in the operating room, venous access was established. Routine monitoring included an electrocardiogram, pulse oxygen saturation (S_P_O_2_), noninvasive systolic blood pressure (SBP), diastolic blood pressure (DBP), and heart rate (HR). After the induction of anesthesia with intravenous fentanyl (3 μg/kg) and propofol (3 mg/kg), a laryngeal mask airway (LMA) (classical type, Tuoren Company, Changyuan, China) was inserted. Subsequently, the lungs were mechanically ventilated with pressure-controlled ventilation. The ventilation parameters were set as a driving pressure of 12–15 cmH_2_O, a respiratory frequency of 14–20 breaths/min, an oxygen flow rate of 2 L/min, the fraction of inspired oxygen was 0.5, an I:E ratio of 1:1.5, and a positive end-expiratory pressure of zero.

The caudal block was performed under general anesthesia in the left lateral position. The D group received 1 ml/kg of analgesic solution that consisted of 0.2% ropivacaine (AstraZeneca Pharmaceutical Company, Beijing, China) and 0.5 μg/kg dexmedetomidine (Jiangsu Hengrui Pharmaceutical Company, Lianyungang, China) for the caudal block. The control group received 0.2% ropivacaine 1 ml/kg alone. The analgesic medications were prepared by the nurses. The driving pressure was adjusted to keep the end-tidal carbon dioxide partial pressure (P_ET_CO_2_) between 35 and 50 mmHg. Anesthesia was maintained with 2%−3% end-tidal sevoflurane to keep the blood pressure within a 20% range of baseline. Anesthetic agents were stopped 5 min before the end of the operation and the children were transferred to the postanesthesia care unit (PACU) at the end of the operation.

The SBP, DBP, and HR were recorded at 5-min intervals during the operation. The time to remove the LMA (extubation time), wake-up time, time to discharge from the PACU, the duration of the caudal block, and the Ramsay sedation scale (RSS) during the first postoperative 24 h were also noted. The adverse events (postoperative agitation, respiratory depression, bradycardia, hypotension, excessive sedation, nausea, and vomiting) were recorded. The LMA was removed when the tidal volumes were >6 ml/kg, the S_P_O_2_ was >96%, and the P_ET_CO_2_ was <50 mmHg during inhalation. The children were discharged from the PACU when the modified Aldrete score was >9. The standards for the modified Aldrete scores were as follows:

- Movements: 2 = spontaneous movement of the arms, legs, and head; 1 = spontaneous movement of the arms or legs with restricted spontaneous head movements; and 0 = no movement of the limbs or head.
- Breathing: 2 = Deep breathing and effective coughing with a normal respiratory rate; 1 = Difficult or restricted breathing but spontaneous breathing is shallow and slow. 0 = Paused or weak breathing that requires assisted breathing.
- Blood pressure: 2 = Within ±20% before anesthesia; 1 = ±20%−49% before anesthesia; and 0 = > ±50% before anesthesia.
- Consciousness: 2 = Completely awake and can answer questions accurately; 1 = the patient can wake up but is drowsy; and 0 = nonresponsive.
- SpO_2_: 2 = air breathing SpO_2_ >92%; 1 = oxygen breathing SpO_2_ >92%; and 0 = oxygen breathing SpO_2_ <92%.

The duration of the caudal block was defined as the time from the caudal injection to the first occasion when the children complained of incisional pain. Respiratory depression was defined as SpO_2_ levels <94% while receiving oxygen and a respiratory frequency of <10 times/min. Hypotension was defined as SBP reduction to >20% from the baseline values and bradycardia was defined as a HR <60 beats/min or reduction to >20% from the baseline values. Children were treated with propofol (1 mg/kg) if postoperative agitation occurred.

The level of sedation was assessed using the Ramsay sedation scale (RSS) (1 indicated that the patient was anxious, agitated, or restless, 2 indicated that the patient was cooperative, oriented, and alert, 3 indicated that the patient was responsive to commands, 4 indicated that the patient was asleep but had a brisk response to a light glabellar tap or loud auditory stimulus, 5 indicated that the patient was asleep with a sluggish response to a light glabellar tap or loud auditory stimulus, and 6 that the patient was asleep and not responsive) (8). The RSS values were recorded at intervals of 1 h during the first postoperative 24 h. Excessive sedation was defined as when the RSS value was >4. Postoperative agitation was defined as an RSS value of 1.

### Statistical Analysis

In this study, the primary outcome was the incidence of postoperative agitation and the secondary outcome was the duration of the caudal block. According to our pilot study, 40 samples in each group were required to allow for dropouts using a two-sided Chi-square test at a significance level of 0.05 with a power of 80%. Data analysis was performed with the SPSS 20.0 statistical software (SPSS Inc., Chicago, IL, USA). Data are presented with mean ± standard deviation. Comparison of the numerical variables between the two groups was performed using a Student's *t-*test for independent samples. The categorical data were compared using a Chi-square test. *P-*values < 0.05 were considered statistically significant.

## Results

**Figure 1:**
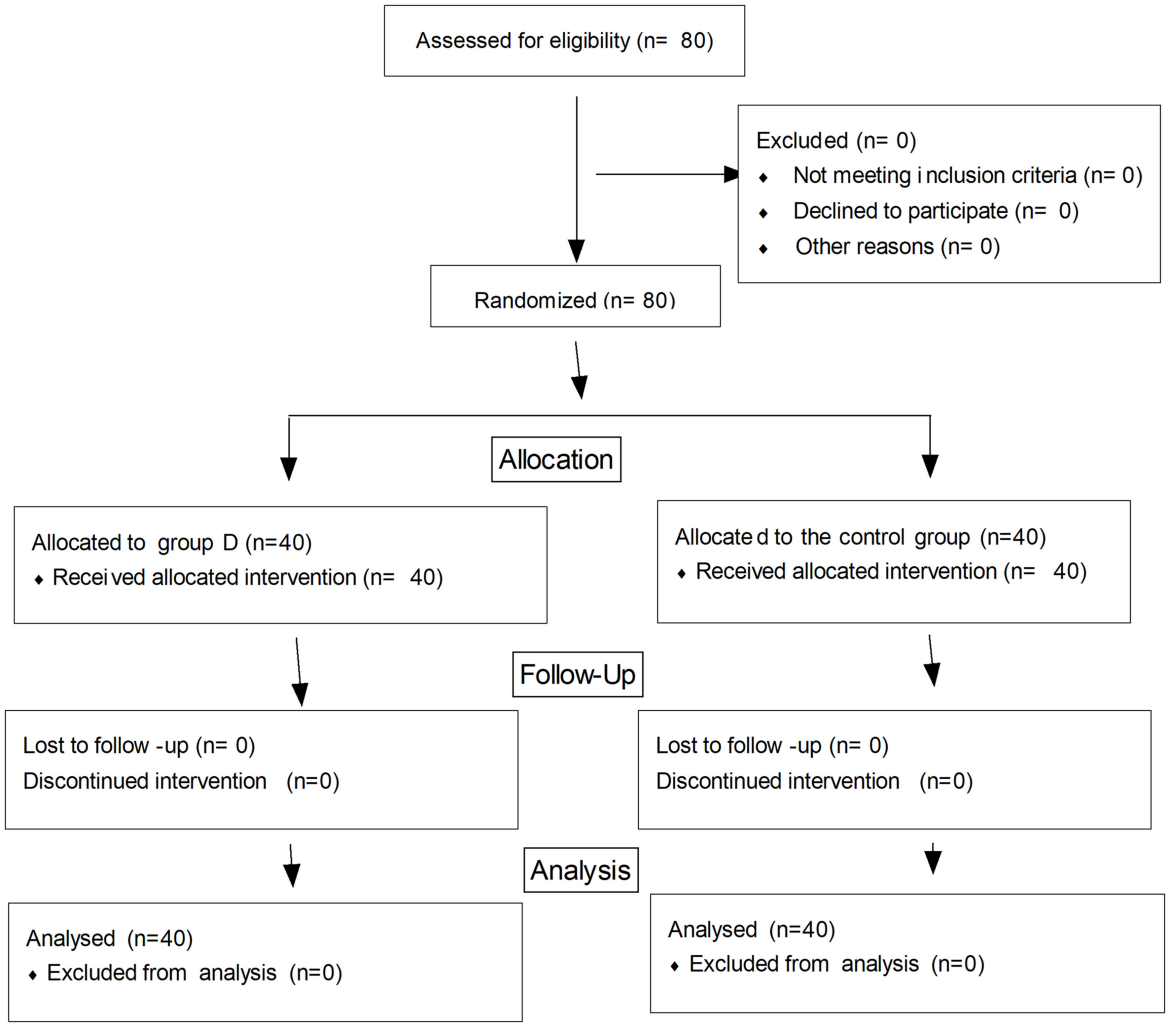
A flow diagram of the study.

**Figure 2:**
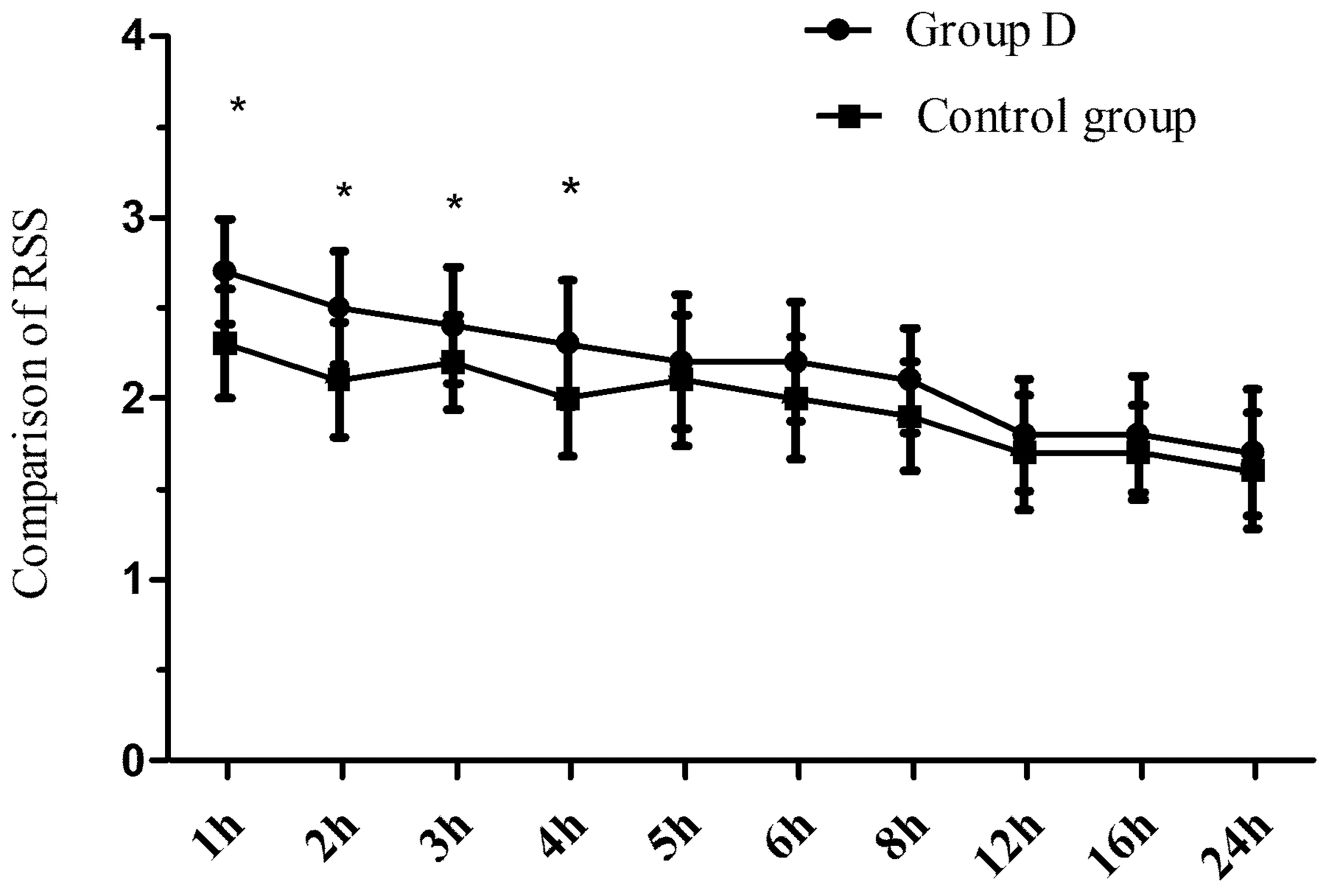
A comparison of the postoperative RSS at different time points, *p < 0.05.

Eighty children were recruited to and completed the study (Figure 1). No significant differences in age, weight, BMI, the duration of operation, and the duration of anesthesia were observed between the two groups (*p* > 0.05) (Table 1). The extubation time was similar between the two groups (8.1 ± 2.0 vs. 8.5 ± 2.1 min, *p* = 0.447), while the time to wake up and discharge from PACU were significantly longer in group D compared with the control group (15.2 ± 2.5 vs. 13.4 ± 1.3 min, 48.2 ± 7.7 vs. 41.5 ± 8.0 min, respectively, *p* < 0.001) (Table 1). The duration of the caudal block was significantly longer in group D compared with the control group (8.8 ± 1.6 vs. 4.6 ± 0.7 h, *p* < 0.001). The postoperative RSS was higher in group D compared with the control group within the first postoperative 4 h but was similar between the two groups during 5–24 h after the operation (Figure 2).

**Table 1:** Data of children (*n* = 40).

**Index**	**Group D**	**Control group**	***P-*value**
Age (year)	3.7 ± 0.9	3.5 ± 1.2	0.466
Weight (kg)	15.2 ± 2.0	14.7 ± 2.2	0.305
BMI (kg/m^2^)	23.6 ± 3.5	23.5 ± 3.2	0.869
Duration of anesthesia (min)	97.6 ± 6.3	98.4 ± 6.3	0.447
Duration of surgery (min)	83.4 ± 7.7	84.3 ± 8.2	0.586
Duration of caudal block (h)	9.7 ± 1.6	4.5 ± 0.7	<0.001
Extubation time (min)	8.1 ± 2.0	8.5 ± 2.1	0.447
Wake-up time (min)	15.2 ± 2.5	13.4 ± 1.3	<0.001
Time to discharge from PACU (min)	48.2 ± 7.7	41.5 ± 8.0	<0.001

*Data are expressed as the mean + standard deviation or number. BMI, body mass index; LMA, laryngeal mask airway; PACU, post-anesthesia care unit*.

There was one case of postoperative agitation in group D, while nine cases were reported in the control group (2.5 vs. 22.5%, *p* = 0.007). There were no significant differences in the incidence of respiratory depression, bradycardia, hypotension, nausea, and vomiting between the two groups. Postoperative hypoxemia and excessive sedation were not observed in either of the groups during the study period (Table 2).

**Table 2:** Adverse events of children (*n* = 40).

**Index**	**Group D**	**Control group**	***P-*value**
Postoperative agitation (*n*)	1(2.5)	9(22.5%)	0.007
Respiratory depression (*n*)	0	0	0.999
Bradycardia (*n*)	2	0	0.494
Hypotension (*n*)	1	0	0.999
Nausea and vomiting (*n*)	1	2	0.999
Excessive sedation (*n*)	0	0	0.999
Postoperative hypoxemia (*n*)	0	0	0.999

*Data are expressed as number (percent)*.

## Discussion

Emergence agitation may cause injury to patients and may also result in the accidental removal of intravenous catheters, dislodgement of urinary catheters, postoperative wound bleeding, and increases in the nursing requirements in PACU. This study indicated that caudal dexmedetomidine prolonged the duration of analgesia and reduced the incidence of postoperative agitation in children undergoing urethroplasty.

The duration of the caudal block was longer in group D compared with the control group. These data indicated that dexmedetomidine prolonged the duration of the caudal block and maintained long-term effective analgesia. Dexmedetomidine can produce analgesia by activating the spinal α_2_ adrenergic receptor (9). In our study, the RSS in the first postoperative 4 h was higher in group D than in the control group, but the RSS was similar between the two groups at 4–24 h after the operation suggesting that dexmedetomidine could increase the sedative effect of the caudal block. Dexmedetomidine can produce a sedative effect by activating the α_2_ adrenergic receptor (10–12). As the sedative effect of dexmedetomidine gradually disappeared, the RSS after dexmedetomidine administration decreased. Hassan et al. (13) reported that caudal bupivacaine combined with dexmedetomidine prolonged the analgesic time of bupivacaine and increased the sedation scores in pediatrics undergoing hypospadias surgery. These observations are in agreement with our findings.

The extubation time (time to remove LMA) was similar between the two groups, but the wake-up time and discharge time from PACU were longer in group D than in the control group. Dexmedetomidine did not cause respiratory depression when used for sedation (14), so it did not result in prolongation of the time to remove LMA. Dexmedetomidine provided lasting sedation and affected the Aldrete score and led to prolongation of the wake-up time and delaying discharge from PACU.

In the present study, the incidence of postoperative agitation was decreased in group D compared with the control group (2.5 vs. 22.5%, *p* = 0.007). It indicated that a single bolus dose of caudal dexmedetomidine 0.5 μg/kg decreased the incidence of postoperative agitation in children undergoing urethroplasty. Postoperative agitation is related to many factors including postoperative pain, the use of inhalant anesthetics, anoxia, the types of surgical procedures, and airway obstruction (15). Postoperative pain and discomfort are the main causes of postoperative agitation. In our study, dexmedetomidine prolonged the duration of the caudal block and maintained long-term analgesia. Excellent analgesia would reduce the incidence of postoperative agitation in pediatric patients. Hence, we concluded that the use of caudal dexmedetomidine at a dose of 0.5 μg/kg reduced the incidence of postoperative agitation in children undergoing urethroplasty. In agreement with our findings, previous studies have shown that the venous infusion of dexmedetomidine decreases the incidence of postoperative agitation in children (1–3).

No significant differences in the incidence of respiratory depression, bradycardia, hypotension, excess sedation, nausea, and vomiting between the two groups in this study were found. Two patients developed bradycardia in group D, but no patients required treatment with atropine. Hypotension and bradycardia are common side effects of neuraxial dexmedetomidine administration. Konakci et al. (16) reported that the hemodynamic adverse events are less pronounced in children compared with adults and may be dose dependent.

## Limitations

Our study has several limitations. Currently, the FDA has not approved the use of neuraxial dexmedetomidine and the levels of pain cannot be accurately assessed in young children (age <6 years). Further studies are needed to assess the side effects of caudal dexmedetomidine.

## Conclusions

This study showed that caudal dexmedetomidine is effective in the prevention of postoperative agitation in children undergoing urethroplasty and prolongs the duration of the caudal block without excessive sedation.

## Data Availability Statement

The raw data supporting the conclusions of this manuscript will be made available by the authors, without undue reservation, to any qualified researcher.

## Ethics Statement

The studies involving human participants were reviewed and approved by the Ethical Committee of Jiaxing Children's Hospital (approval number: 2018-36, Chairman: Prof L. Xia). Written informed consent to participate in this study was provided by the participants' legal guardian/next of kin.

## Author Contributions

WeZ, JH, and MS: study design and data analysis. WaZ: patient recruitment and data collection. JS and WaZ: writing of the paper. All authors contributed to the article and approved the submitted version.

## Conflict of Interest

The authors declare that the research was conducted in the absence of any commercial or financial relationships that could be construed as a potential conflict of interest.

## Publisher's Note

All claims expressed in this article are solely those of the authors and do not necessarily represent those of their affiliated organizations, or those of the publisher, the editors and the reviewers. Any product that may be evaluated in this article, or claim that may be made by its manufacturer, is not guaranteed or endorsed by the publisher.
